# Biomechanical evaluation of type p condylar head osteosynthesis using conventional small-fragment screws reinforced by a patient specific two-component plate

**DOI:** 10.1186/s13005-020-00236-0

**Published:** 2020-10-19

**Authors:** Tetiana Pavlychuk, Denis Chernogorskyi, Yurii Chepurnyi, Andreas Neff, Andrii Kopchak

**Affiliations:** 1 Department of Stomatology, O.O. Bogomolets National Medical University, Kyiv, Ukraine; 2 Department of Oral and Craniomaxillofacial Surgery, UKGM GmbH, University Hospital Marburg and Philipps University Marburg, Marbug, Germany

**Keywords:** Condylar head fracture, Reinforcement plate, Screw osteosynthesis, Biomechanics, FEA, Finite element analysis

## Abstract

**Background:**

The aim of this study was to evaluate via finite element analysis (FEA) the biomechanical behavior of conventional small-fragment screws reinforced by a patient-specific plate in type p condylar head.

**Methods:**

A finite element model of the mandible was created using Mimics 12.1 software. A type p condylar head fracture was simulated in the right condyle, and the left condyle was used as a control. Two patterns of fixation were investigated: conventional two-screw fixation and the same fixation system reinforced with a small, patient-specific plate. Surface models were imported into the software Ansys 5.7for further volume mesh generation.

**Results:**

The highest stress gradients were observed in the cortical layer of the lateral fragment, located near the screw. The conventional fixation method resulted in equivalent stresses 2 to 10 times greater than the reinforced method. Rigidity of fixation in the reinforced method increased up to 1.25–3 times compared to the conventional two-screw technique.

**Conclusion:**

This study’s findings suggest significant benefits in unfavorable biomechanical conditions from reinforcement of the standard two-screw fixation of condylar head fractures with a small, patient-specific plate acting as a washer.

## Background

Condylar head fracture management is still one of the most controversial issues in maxillofacial surgery [1–3]. A recent randomized multicentre study reported that open reduction and internal fixation (ORIF) resulted in better morphologic and functional outcomes compared with non-surgical treatment [1]; according to a recent long-term follow-up study, it also leads to a better quality of life [3]. ORIF facilitates anatomically accurate repositioning of the dislocated fragments and restoration of the ramus to its normal height, which is important to avoid mandibular movement restriction, malocclusion or temporomandibular joint (TMJ) internal derangement [2–5]. Several studies have shown that the main predictors of unfavorable functional prognosis at long term follow up are adequate reconstruction of the condylar head and minimally invasive revision of the surrounding soft tissues [2, 3, 6–8]. At the same time, stable ORIF of condylar head fractures remains a significant challenge for surgeons due to limited access and visualization, complex individual trauma patterns, specific anatomy and biomechanical relations of the injured area [6, 8].

Numerous surgical procedures and fixation devices (either metal or polymeric) for condylar head fractures have been proposed and studied. Different authors have reported on the application of micro- and miniplates, lag screws, cannulated screws, small-fragment positional screws, bioresorbable pins and screws, each with varying rates of success [9–14]. According to the literature, the standard technique for internal fixation of condylar head fragments is titanium screw osteosynthesis via the lateral end of the ascending ramus [13, 15]. Due to the complex loading conditions of the TMJ in normal mastication, at least two fixation screws are necessary to compensate for rotational and share stresses [9, 11, 12].

The application of two-screw fixation for dia capitular condylar head fractures was first described by Rasse et al. and later modified by Neff et al., who improved the technique and proved its efficacy in clinical and biomechanical studies [3, 12, 13, 16]. This method(i.e. 1.7–1.8 mm small-fragment positional screw osteosynthesis (SFPSO))is minimally invasive, anatomically based, and has a number of advantages over the alternative methods of mini- or microplates, lag screws and bioresorbable pins. The main problem facing surgeons in application of this technique is to ensure the correct repositioning of the fragments and to keep them in reduced position during the screw installation. This can be quite difficult because of limited surgical access and bad visualization, especially in cases of “comminuted” (viz. major fragmented) fractures or butterfly fragmentation of the cortex in the lateral pole area or at the posterior surface of the condylar head with loss of the main anatomical landmarks. Temporary screws [12] or titanium microplates are often used as a kind of pre-fixation device [17] for precise adjustment of the main fragments and maintaining their reduced position during drilling and insertion of long positional bi- or monocortical screws. These ancillary plates and screws are usually removed before wound closure, but they occasionally remain in place for reinforcement of the fixation system or to fix butterfly fragments.

The finite element analysis performed by Kozakiewicz and Xin demonstrated that, under functional loading, the highest stress concentration is observed within the cortical layer of the lateral bony segment and posterior condylar surface, near the screw holes [9, 11]. The stress gradients may increase in cases with a thin cortical layer or comminution of the condylar head’s lateral pole resulting in cortical bone destruction, cracks, fragmentation and even the failure of the whole system. The use of reinforcement plates may be beneficial in such cases due to a more even load distribution in the fractured area and an increased stability of the fixation system. However, excepting the report on the infrequent clinical use of these ancillary microplates as published by Kolk and Neff, we could not find any specific research, either experimental or clinical, on the efficacy of a reinforcement plate combined with two conventional small-fragment screws in condylar head fractures [3].

Recently, computer-aided design (CAD) and computer-aided manufacturing (CAM) have been successfully used in maxillo-facial traumatology and reconstructive surgery [18]. CAD/CAM technology provides opportunities for surgeons to: simulate the operation on a computer, perform virtual fragment repositioning, select the appropriate method of fixation, and increase the precision of surgical manipulations by manufacturing surgical guides or patient-specific implants. Surgical guides for proper repositioning and fixation as well as patient-specific plates have been successfully used by Suojanen J, Chepurnyi, and Yang in orthognathic surgery and orbital, midfacial and mandibular reconstruction [19–21]. Several authors have reported that CAD/CAM technology enabled significantly shorter operating times, lower operation risks, and a more precise fit and better stability for the bone-fixator system [20–22]. In condylar head fractures, CAD has so far been used by Wang, Yang, Smolka and Han for precise virtual repositioning of the condylar fragments, decision-making about the appropriate type, length and angulation of the screws, and estimation of the possible operative risks [15, 21, 23, 24]. As accurate reduction and fixation are key steps during surgery, seeking an effective method to increase this accuracy has been a point of discussion in recent years [24]. Nevertheless, the literature to date has not reported on surgical guides or patient-specific fixators for condylar head fractures or high condylar neck fractures. To make such a tiny construction, which can be applied via a very limited surgical access with sufficient mechanical properties, specific design and complex manufacturing processes are necessary.

We have designed a two-component patient-specific titanium guide to ensure anatomically correct reduction of the condylar head fragments as well as an appropriate positioning and angulation of the positional small-fragment fixation screws. The guide is designed to help correctly reposition small fragments in three dimensions and then to hold the fragments in a reduced position during the insertion of fixation screws. Having achieved these goals, the guide can then be partially removed in order to avoid intracapsular scarring [13]. The remaining modular component – a small, individualized plate located at the lateral surface of the condyle – stays extracapsular and is used for reinforcement of the fixation system in unfavorable biomechanical conditions.

## Methods

The aim of this study was to evaluate the biomechanical behavior of conventional small-fragment screws reinforced by apatient-specific plate in type p condylar head fractures (i.e. fractures within or lateral to the pole zone, according to AOCMF Classification System [25]) using finite element analysis.

### Design of the patient-specific two-component plate

**Fig. 1:**
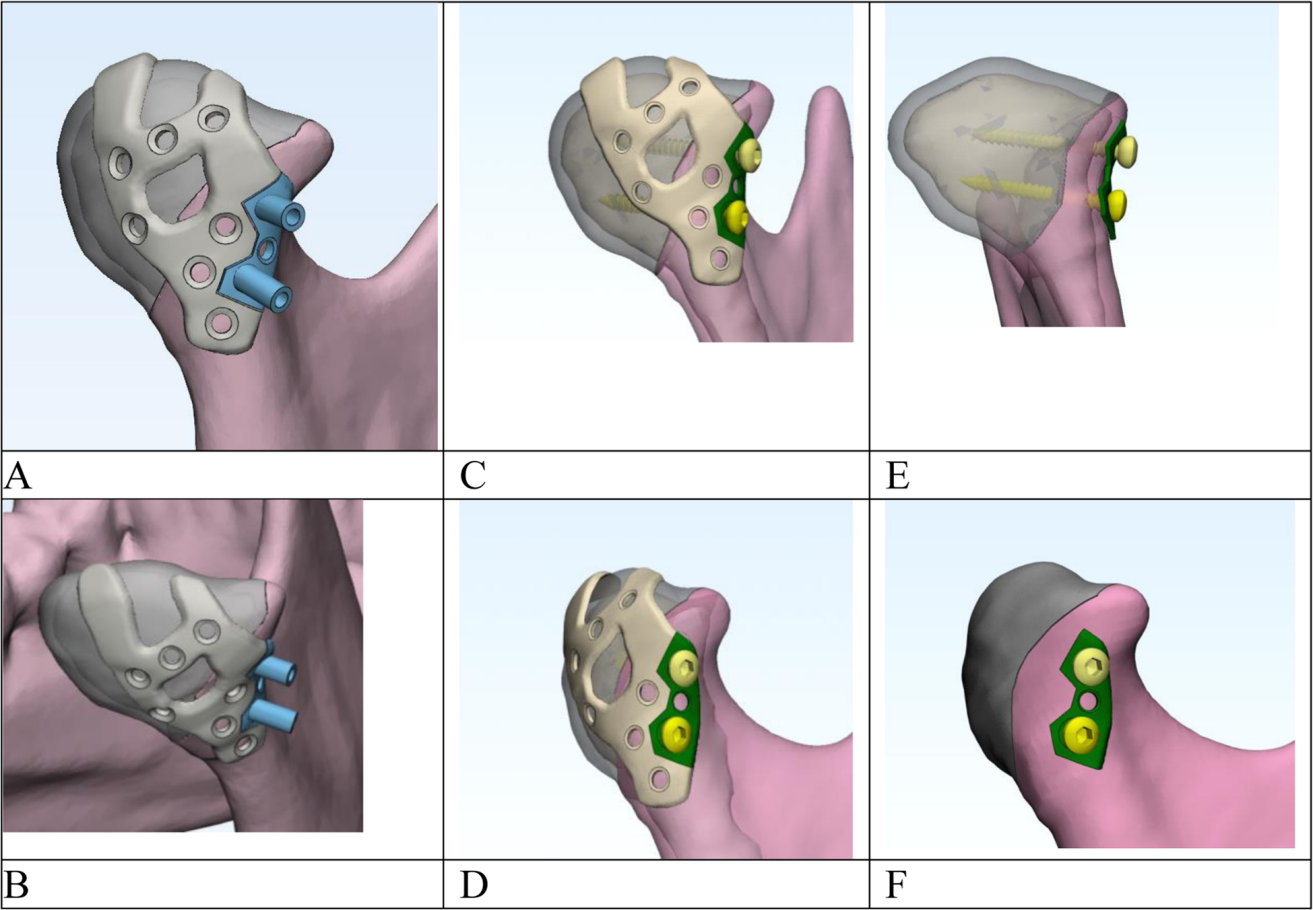
Design of the patient-specific titanium plate and surgical guide for reduction and reinforcement of the conventional two-screw fixation system. **a, b** – the first component of the plate for proper reduction of the condylar head with surgical guide for positioning of long screw, **c,d** - the first component of the plate and PSRP with two small-fragment positional screw, **e, f** - PSRP with two small-fragment positional screw with removed first component (final view of fixation system)

The conceptual design of the titanium patient-specific reinforcement plate (PSRP), to be used for reduction and for reinforcement of the conventional screw fixation system, was developed on 3D models of a mandible with dislocated condylar head fractures. Following virtual reduction of the bone fragments in CAD software, models of the two-component sectional plates were created using a geometric approach according to protocols detailed by Parr et al. [26]. The first component of the plate was designed to ensure proper reduction of the condylar head. This component was virtually warped to fit the morphology of the bone surface after appropriate repositioning of all bony fragments. The main aspects that were considered in individual modelling of this component were: viability of plate insertion and fixation via a conventional retroauricular approach, anatomical ‘safe zones’ (attachment points for the lateral pterygoid muscle, capsule and ligaments, which need to be preserved), fracture pattern and the type of fragmentation. The second component, the small reinforcement plate, was modelled at the lateral surface of the condylar ramus, with two bigger holes (1.8 mm in diameter) for the small-fragment positional screws. The third component, the surgical guide, was modelled to facilitate appropriate positioning and respective optimum angulation of the long, or positional, screws. The surgical guide is identical to the reinforcement plate with the addition of two positioning holes for the screws. After drilling, the surgical guide can be removed and the reinforcement plate can be fixed in the same position. The edge of this plate, including retention elements, is exactly matched to the edge of the first component of the construction. The thickness of the bars and retention elements in both components is at minimum 0.6 mm, which was necessary for manufacturing the plate using direct metal laser sintering. A number of small holes were modelled on both parts of the plate for fixation to the medial and lateral fragments via microscrews. After reduction and internal fixation of the fracture, the microscrews and the first component of the plate can be removed, while the second component remains, functioning as a washer to reinforce the positional screws in cases with fragmentation of the condylar head’s lateral pole or a thin cortical layer of the largest fragment (Fig. 1). Consequently, only the second component (reinforcement plate) was considered in the following biomechanical analysis.

### Construction of 3D models

A three-dimensional virtual model of the mandible for FEA was constructed in Mimics 12.1 software (Materialise, Belgium), based on a multispiral computed tomography (CT) data set of a patient’s intact mandibular bone of normal anatomical shape and dimension. The CT was obtained using a Toshiba Aquilon 16 scanner with slice thickness 0.5 mm. The DICOM data were imported into Mimics 12.1 software for segmentation. Separate “masks” were generated and modified for either the cortical or spongious layer of the mandible. The cortical layer at the area of the condylar head was thinned to a maximum of 0.9 mm. Three-dimensional surface objects were generated from each mask and remeshed to improve their quality. The right condyle of the mandible was used for simulations, while the left one remained intact and served as a control. The right condylar head was cut using Mimics’polyplane tool to reproduce a typical type p condylar head fracture, which is synonymous with a type B fracture, according to Neff et al. and Loukota et al. [13, 25, 27]. The cutting plane ran through the lateral pole of the condylar head and continued to the medial side of the condylar neck. Medial and lateral fragments were fully separated; a gap of 0.25 mm was created between the fragments to exclude the effect of bony contact. Three-dimensional surface models were also developed for conventional small-fragment positional screws (length 13 mm, diameter 1.8 mm) as well as the patient-specific plate using the CAD software Autodesk Inventor (Autodesk, Inc., San Rafael, CA). Surface models were assembled using Boolean operations. Two patterns of fixation were investigated: 1) conventional long, or positional, two-screw fixation where, according to recommendations by Neff et al., screws were inserted through the medial and lateral segments perpendicularly to the fracture line,^3^and 2) the same fixation system reinforced by a small, patient-specific plate (0.6 mm thickness), where the positional screws were inserted in the same position through the holes of the plate. These surface models were imported into the software Ansys 5.7 for further volume mesh generation.

### Mesh generation and materials properties

**Fig. 2:**
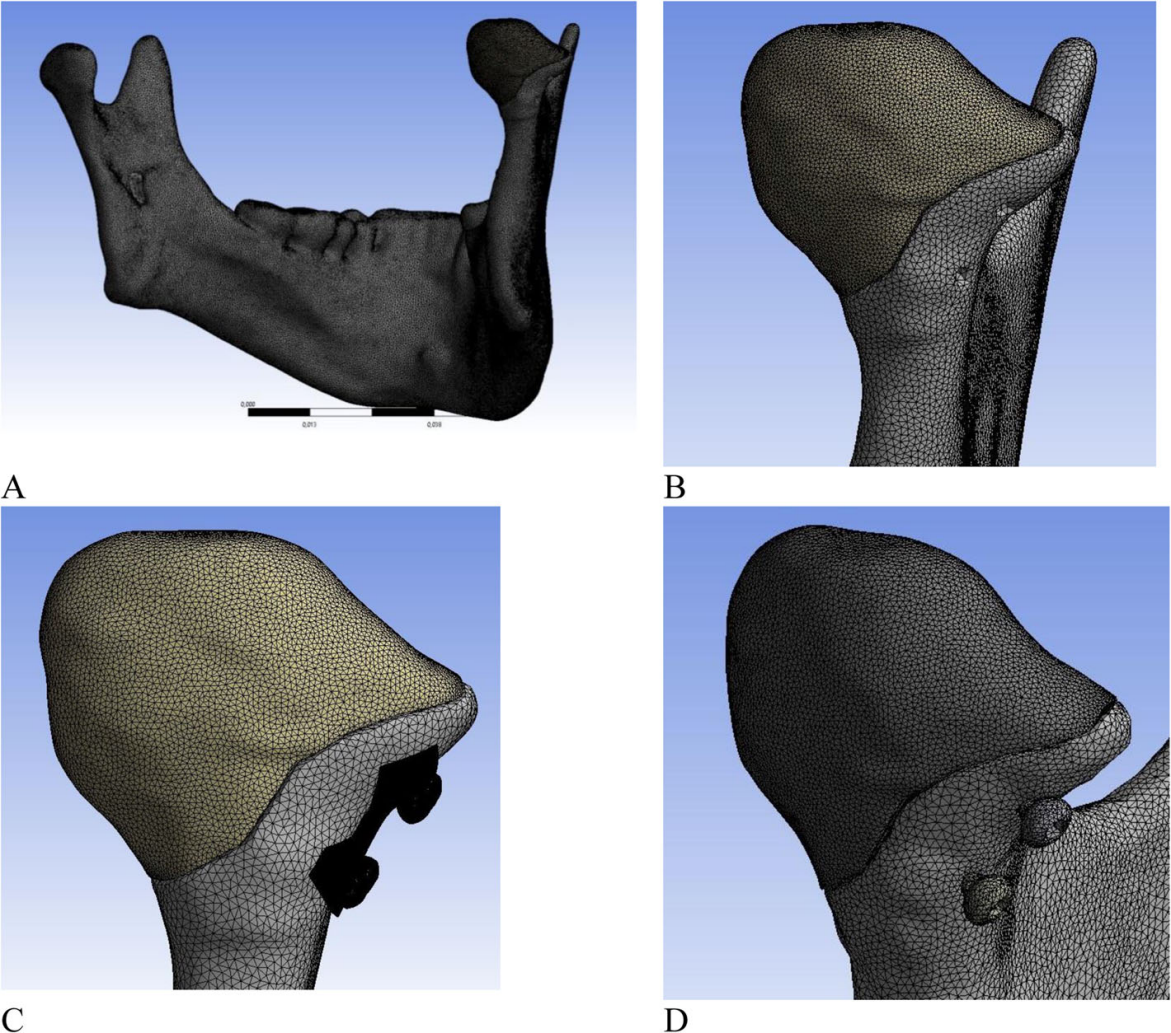
Finite element model of the mandible after condylar head osteosynthesis: **a** – general view, **b** – condylar head with fixators hidden, **c** –condylar head fixed with 2 screws and PSRP, **d** – condylar head fixed with 2 screws

Preprocessing of the models was performed in Ansys 5.7 software (Swanson Ansys Inc., Houston, PA, USA). Separate volumes were created and meshed for each segment, followed by assignment of material properties. The optimal mesh size was determined using a convergence test. The number of tetrahedral finite elements in the generated volume mesh was 3,915,059 for the two-screw model and 6,378,137 for the PSRP model, the number of nodes was 698,137 and 1,152,636, respectively. These numbers are sufficient to reflect the individual geometry of the mandible while still being manageable for numerical simulation on a conventional computer (Fig. 2).

The basic mechanical properties of titanium hardware and bone were referenced from previous research [9]. For numerical calculation simplification, the bone tissue was defined as solid, homogeneous (within one material type), linearly elastic and isotropic, as in previously published protocols. The Young’s modulus of elasticity for the cortical layer was 13 GPa, in accordance with experimental data. The Young’s modulus of spongious bone was 0.8 GPa. Poisson’s ratio ν was considered 0.3 for all elements of the bone. The typical properties of titanium alloy (Ti-6Al-4 V, Grade 5) were assigned to the screws and patient-specific reinforcement plates (E = 114GPa, ν = 0.34). The morphological and mechanical peculiarities of the periodontal ligament and dento-alveolar complex were deemed irrelevant and ignored in generated models. As the morphology of these structures is very complex and their mechanical properties are not well studied, simplification of the model reduced the number of inadequacies and errors in numerical solution and results postprocessing.

### Loading and boundary conditions of the FE models

**Fig. 3:**
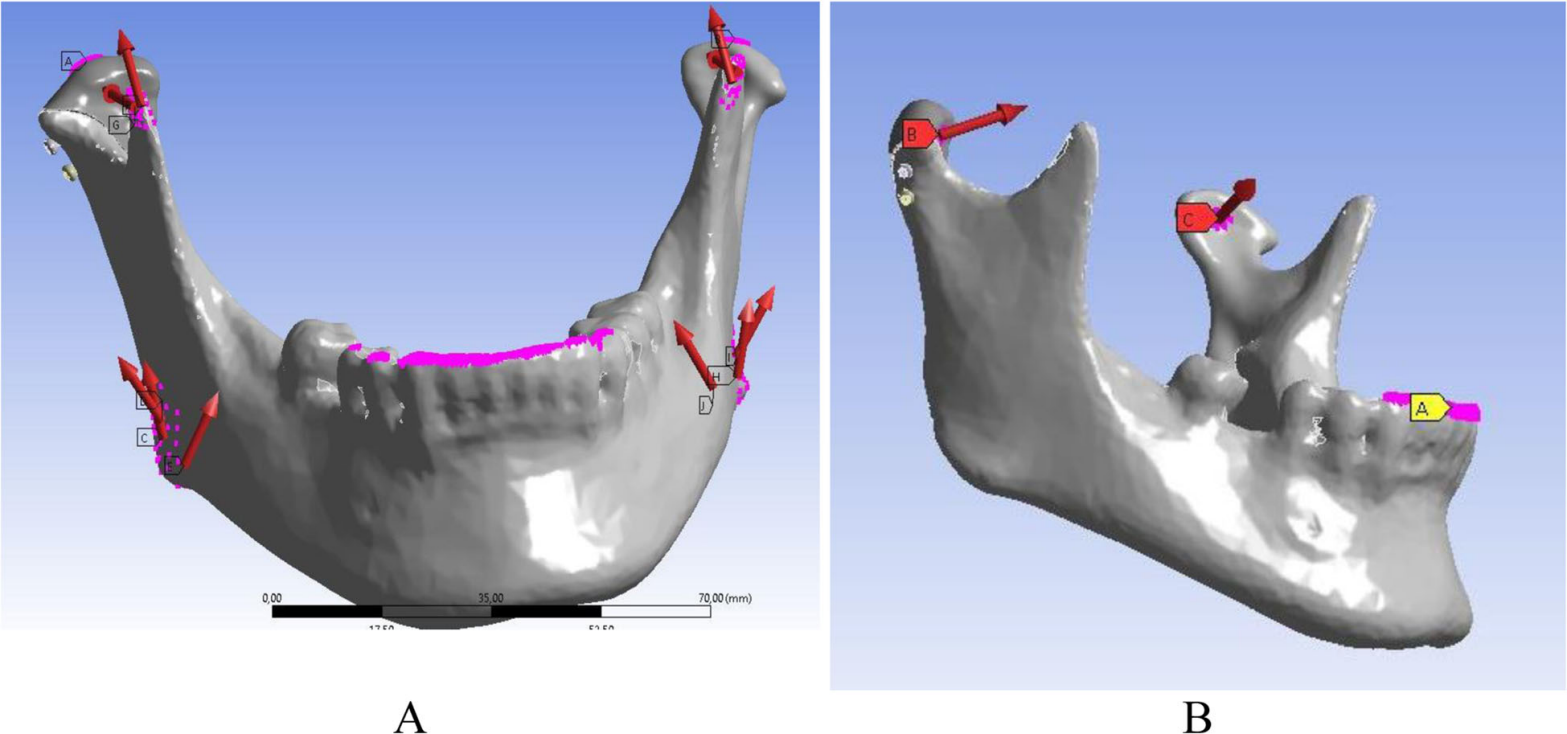
Muscular forces and constraints applied in finite element simulation. **a**– constraints were applied to the articular surface of the condyle and occlusal surface of the incisors and canines, force –simulation of inferior lateral pterygoid muscle contraction; **b** –constraints were applied to the occlusal surface of the incisors and canines, force –simulation of medial pterygoid, superficial and deep masseter, anterior and posterior temporalis contraction

Two loading conditions were applied in the models in this study. In the first, loading condition constraints were applied to the occlusal surface of the incisors and canines with zero degrees of freedom, and the inferior lateral pterygoid muscle was modelled, simulating protrusion of the mandible (Fig. 3a).

In the second condition, applied to the loading of anterior biting contraction of 5 pairs of muscles medial pterygoid, superficial and deep masseter, anterior and posterior temporalis – was simulated. Muscular forces were applied to the nodes representing the anatomical regions of muscle attachment (Fig. 3b); their values and directions were set according to experimental data published by Korioth TW and Koolstra (Table 1) [28–30]. Constraints were applied to the condylar articular surface and occlusal surface of the incisors and canines with zero degrees of freedom (both condyles were fixed in all translations and rotation).

**Table 1:** The muscular force applied in the FE models according to experimental data by Korioth and Koolstra

Side	Direction	Muscular force (N)					
		SM	DM	ILP	MP	AT	PT
	**Force (maximum loading)**	272.8	69.8	112.8	240	308	222
**RS**	Fx	−56.56	−38.19	71	116.6	46.4	44.4
	Fy	−114.2	−25	−85	− 189.67	−14.6	−192.4
	Fz	240.9	53	19	89.56	305.5	103.6
**LS**	Fx	56.56	38.19	− 71	− 116.6	−46.4	44.4
	Fy	−114.2	−25	−85	−189.67	14.6	192.4
	Fz	240.9	53	19	89.56	305.5	103.6
	Force **(normal loading)**	68.2	17.45	28.2	60	77	55.2
**Rs**	Fx	−14.14	−9,5	17.75	29.16	11.61	11.1
	Fy	−28.55	−6.25	−21.37	−22.39	−3.6	−48.1
	Fz	60.23	13.25	4.93	47.41	76.38	25.9
**LS**	Fx	14.14	9.5	−17.75	−29.16	−11.61	11.1
	Fy	−28.55	−6.25	−21.37	−22.39	3.6	48.1
	Fz	60.23	13.25	4.93	47.41	76.38	25.9

In both models, muscular force was reproduced at normal mastication (25% of maximal contraction force) and at maximal muscular contraction to estimate the differences in stress and strain distribution between the methods of fixation in various loading conditions.

Contact between the screws and the plate, as well as between the screws and the bone, were considered as bonded. A linear static solution was generated in all cases. After solving each model, calculations were performed to find the total deformation, maximal principal strain, and patterns of stress and strain distribution. The maximal Von Mises stresses were also recorded and visualized in a color gradient scale for both cortical and spongious layers and for titanium hardware.

## Results

### Rigidity of fixation

The volume of the two titanium small-fragment screws used for fixation of the condylar head was 40.9 mm^3^. The volume of the patient-specific plate was 19.2 mm^3^. Thus, the overall volume of the titanium hardware for PSRP fixation was 32% higher than in the conventional method.

**Fig. 4:**
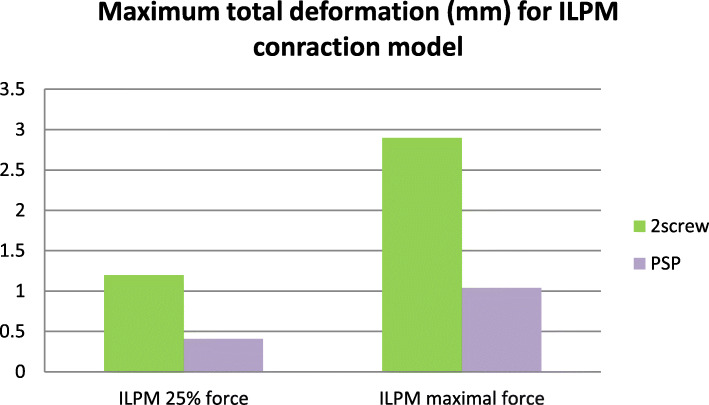
Maximal total deformation for ILPM contraction

**Fig. 5:**
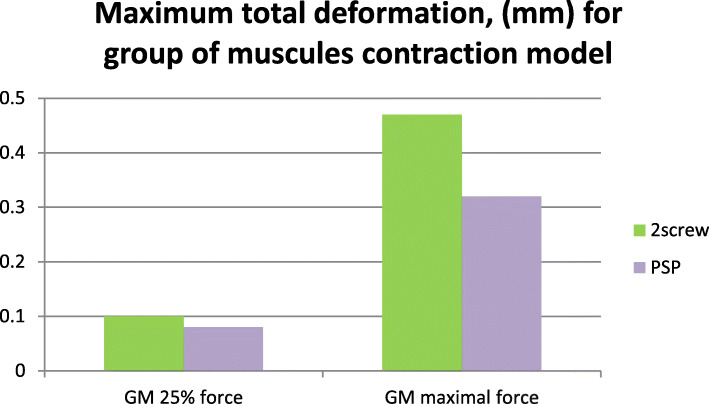
Maximal total deformation for anterior biting load

The FEA proved the adequate rigidity of both fixation systems in normal mastication and even at maximal contraction of the muscles, reproduced in the models. Rigidity was characterized by the total deformation value (i.e. the maximum displacement in mm of the fixed points under functional load) and appeared to be 12–20% less than in the intact, contralateral condyle. Maximal value of the total deformation at the fracture line for different fixation methods is presented in Figs. 4 and 5.

At maximal contraction of the lateral pterygoid muscle, deformationwas2.9 mm in conventional modelsand1.4 mm in PSRP models. In anterior biting, the differences were less significant: total deformation was 0.47 mm and 0.32 mm, respectively. In displacement contours produced at normal mastication loading (25% of maximal contraction force), the total deformation was 1.2 mm vs 0.41 mm for contraction of the lateral pterygoid muscle and 0.1 mm vs 0.08 mm for anterior biting.

### Stresses and strains in the hardware

**Fig. 6:**
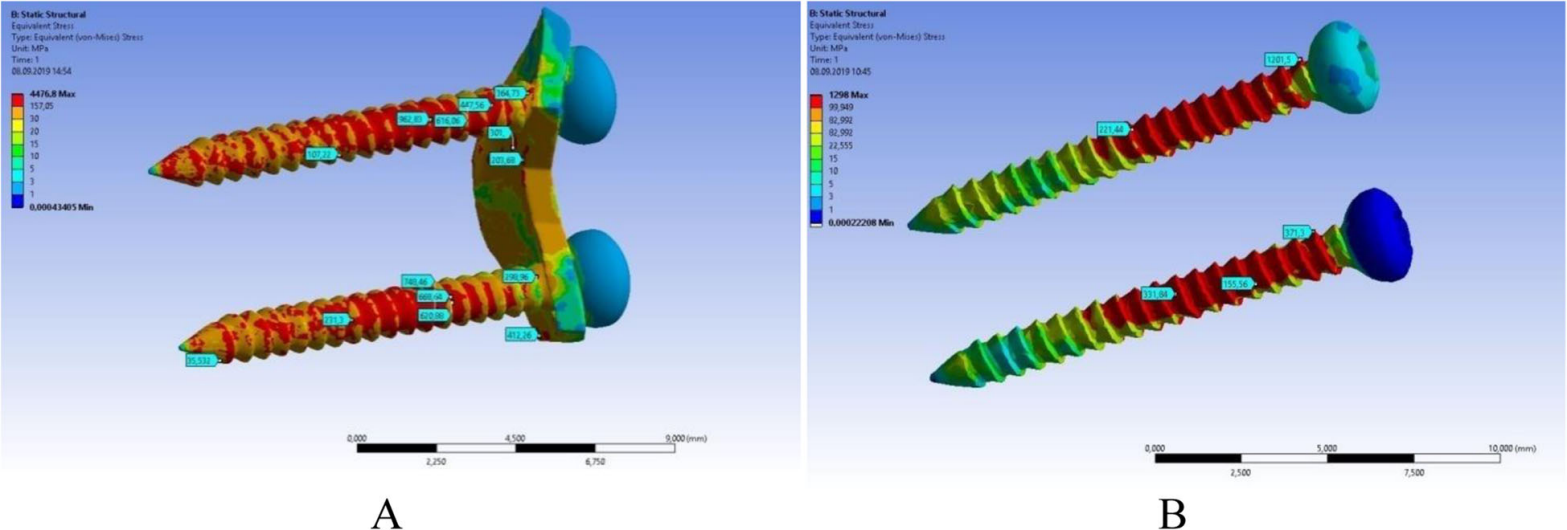
Von Mises stresses (MPa) in titanium hardware under lateral pterygoid muscle maximal contraction loading. **a** - PSRP fixation system, **b** - conventional fixation system

Breakdown, in this case significant non-reversible plastic deformations of the screw fixators used for condylar head fractures, is not commonly seen in clinical practice. However, as breakdown may lead to bone resorption, this study analysed the stress and strain states of the titanium hardware. The main parameter used for prediction of the hardware’s mechanical behavior was the von Mises stress values. In all models, the highest stress was found at the thread terns located nearest to the fracture line. All loading conditions saw high stress values in the upper screw. In normal mastication (25% force), the stress values never exceeded the ultimate stress value for Grade 5 titanium (850–1100 MPa). However, for maximal contraction of the lateral pterygoid muscle, the stress value increased up to 1218 MPa for conventional fixation and up to 962 MPa for the PSRP fixation system. Peak stresses were observed in the small areas of the thread edges, but the surrounding areas and cross-sections of the titanium hardware saw significantly decreased stress. Inside the patient-specific plate, the von Mises stress values were always lower than in the screws and never exceeded 450 MPa (Fig. 6).

**Fig. 7:**
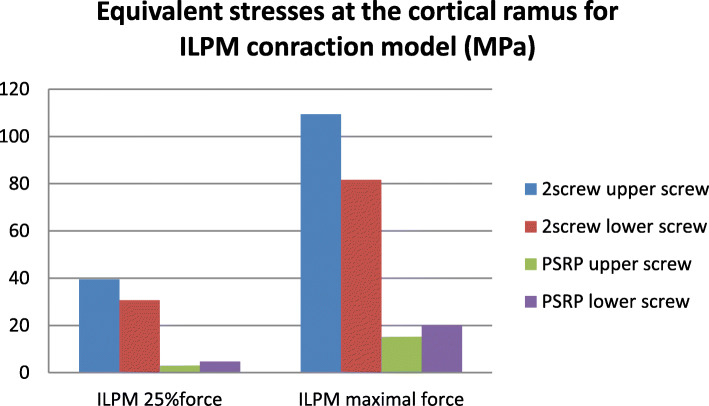
Maximal von Mises equivalent stress in the cortical bone for inferior lateral pterygoid muscle contraction

### Stresses and strains in the bone

**Fig. 8:**
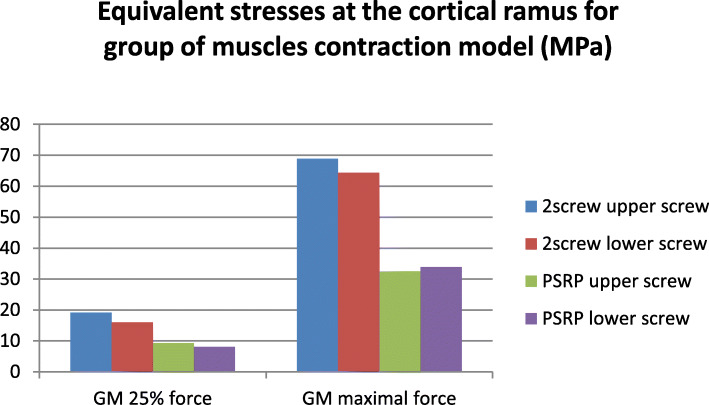
Maximal von Mises equivalent stress in the cortical bone for inferior anterior biting load

**Fig. 9:**
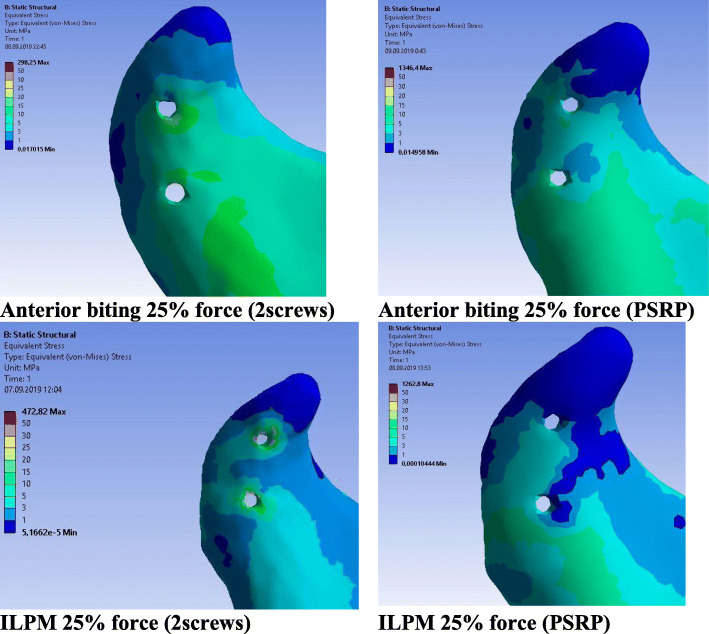
Von Mises equivalent stress gradients in the cortical bone for normal mastication loading. **a** - anterior biting contraction model with 2 screw fixation, **b** - anterior biting contraction model with PSRP fixation system, **c** - inferior lateral pterygoid muscle contraction model with 2 screw fixation, **d** - inferior lateral pterygoid muscle contraction model with PSRP fixation system

**Fig. 10:**
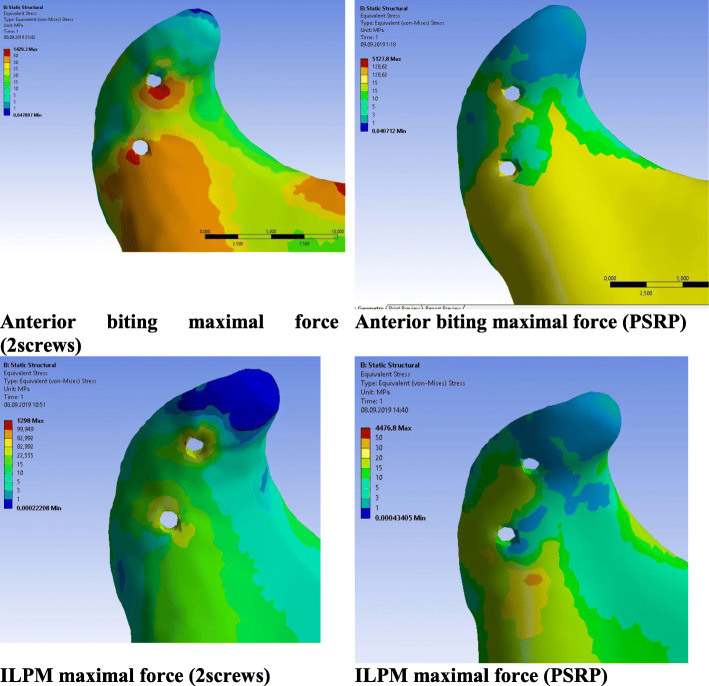
Von Mises equivalent stress gradients in the cortical bone for maximal muscular contraction loading **a** - anterior biting contraction model with 2 screw fixation, **b** - anterior biting contraction model with PSRP fixation system, **c** - inferior lateral pterygoid muscle contraction model with 2 screw fixation, **d** - inferior lateral pterygoid muscle contraction model with PSRP fixation system

In the normal condyle, the stress state was characterized by even distribution, with maximal concentration in the lateral and posterior surfaces of the condylar head and neck. In all models, the maximal stress value of the intact condyle was less than 30 MPa, even when maximal muscular contraction forces were applied. On the injured side, the stress concentration areas were located near the screw holes in the lateral or medial bone segments. The highest stress gradients were observed in the cortical layer of the lateral fragment and reached their highest values in the models with maximal contraction of the lateral pterygoid muscle. While the reinforcement plate did not cause a major decrease in stress values in the titanium hardware, its influence on the bone was much more significant. The equivalent stresses in the conventional fixation method were from 2 to 10 times greater than those in the PSRP method. The most pronounced load-bearing effect of the reinforcement plate was observed in ILPM models; where the maximum equivalent stress in the cortical bone around the upper screw was 109.4 MPa for conventional fixation, it was 15.2 MPa for the reinforced system. For the second screw, the highest stress values were 81.7 MPa and 20 MPa, respectively (Figs. 7, 8, 9 and 10). All models demonstrated high equivalent stress values in the spongious bone of both the medial and lateral segments, located near the screw holes. Stress values were higher in anterior bite loading, reaching 40 MPa at maximal contraction force of the muscles. The reinforcement plate did alleviate spongious bone stress values by 10–60%, but the decrease was less significant than in the cortical layer.

In previous studies, it was found that the most reliable predictor of biomechanical behavior in bone and its reactions to mechanical load is the maximum principal strain [31, 32]. Table 2 presents the values of this parameter according to each applied method of fixation inside the cortical bone near the fixation screws. As shown, conventional two-screw fixation (compared with the reinforced system) was associated with significantly higher strain values in the cortical bone near the screw holes.

**Table 2:** The maximum principal strain in the cortical bone near the screw holes, depending on the fixation method used

	2 Screw		PSRP	
	Upper screw	Lower screw	Upper screw	Lower screw
**ILPM 25% force**	1.8 × 10^−3^	2.2 × 10^−3^	0.17 × 10^− 3^	0.35 × 10^− 3^
**ILPM maximal force**	9.4 × 10^− 3^	0.095 × 10^− 3^	0.91 × 10^− 3^	1.1 × 10^− 3^
**GM 25% force**	1.3 × 10^− 3^	0.73 × 10^− 3^	0.34 × 10^− 3^	0.6 × 10^− 3^
**GM maximal force**	5.2 × 10^− 3^	4.7 × 10^− 3^	1.2 × 10^− 3^	2.5 × 10^− 3^

*SM* superficial masseter, *DM* deep masseter, *MP* medial pterygoid, *ILP* inferior lateral pterygoid, *AT* anterior temporalis, *PT* posterior temporalis

## Discussion

The management of condylar head fractures has been associated with considerable controversy over the years. However, the latest clinical studies have clearly shown that open reduction and internal fixation (ORIF) offers favourable functional outcomes for this type of mandibular trauma. For condylar trauma in general (i.e. base and neck fractures), Worsaae and Thorn, Neff, Ellis and others have demonstrated that surgical treatment provides better results than conservative management in adult patients [6, 33, 34]. Properreduction and rigid fixation of the bony fragments was impossible in most of the conservatively treated patients, resulting in functional deficits and compromised quality of life [34]. Current trends in maxillo-facial traumatology are associated with an increased number of patients treated with ORIF and extensive research aiming to optimize surgical techniques and fixation hardware [6].

At the same time, surgical treatment for condylar head fractures remains challenging and non-surgical treatment has significant limitations and drawbacks, as reported by Hlawitschka M [2, 35]. When ORIF is used to treat such fractures, the appropriate fixation method as well as the type, material, number, dimension and geometric shape of fixation devices and elements are important points of discussion.

The clinical and biomechanical simulations performed by Neff et al., revealed that, for condylar head fractures, the use of two or three small-fragment positional screws is superior other methods of osteosynthesis, such as mini- or microplates. Screw fixation provides better fixation stability and avoids excessive loading of the bone tissue [12]. It also preserves the attachment of the lateral pterygoid muscle and capsular ligaments, reduces articular scarification and ensures sufficient blood supply to the condyle. According to Kolk and Neff, ORIF leads to significantly reduced loss of mandibular vertical height and provides superior functional outcomes compared to the use of mini- and microplates [3].

Conventional two-screw fixation is still technically challenging due to poor visualisation and limited access to the fracture area. Each surgeon handles the fractured fragments according to their own clinical experience, which frequently results in improper reduction and screw positioning, followed by development of complications and even an impaired functional prognosis. The most problematic cases are fractures with fragmentation of the posterior surface and the lateral pole of the condylar head, leading to the loss of anatomical landmarks. In these cases, repositioning of the fragments becomes less predictable. Additionally, due to screw installation, the torque values may be too high to keep the fragments in the correct position. Another problem is that, in cases when the lateral condylar segment (or its cortical layer) is not thick enough (e.g. vertical fracture patterns, Neff et al.), the positional screws cannot effectively stabilize mastication forces, resulting in bone overloading, fixation failure, and loosening or translocation of the titanium hardware [25].

We have developed the concept of a patient-specific device for guided TMJ surgery with the use of advanced CAD/CAM technology in order to increase the accuracy of fragment reduction and to reinforce fixation, especially in the complex cases noted above. The proposed two-component patient-specific titanium guide and small individualized plate located at the lateral surface of the condyle, can be used both for precise fragment reduction and screw positioning and for reinforcement of the fixation system.

The use of CAD/CAM technology in condylar head fractures has been previously reported by Wang, Yang, Smolka, and Han [15, 21, 23, 24]. However, there are few articles devoted to this problem; all existing literature is limited to direct measurements and virtual simulation of the surgical procedures in CAD software. Though the surgical procedure itself remains highly operator-dependent, computer modelling provides a wealth of additional information either about the trauma pattern and anatomical parameters of the injured zone or reference data about position and angulation of the screw hole.

The existing clinical research gives evidence that the precision and predictability of surgical intervention in orthognathic, bone reconstructive and traumatological surgery, including the management of base and neck fractures, is significantly increased by the additional manufacturing and application of patient-specific plates or implants, as well as the implementation of surgical guides for bony fragment reduction and installation of plates and screws [19–21, 36]. The introduction of a patient-specific plate facilitates virtual operation planning and reduces the deleterious impact of low operator skill and experience. However, we could not find any reference in the literature that such an approach has been used for condylar head fractures. The design proposed in this study meets the demand for such a construction. It can be applied during the conventional (e.g. retroauricular) approach, and its dimension is adapted to the limited surgical access to the mandible; its shape is determined by existing anatomical ‘safe zones’ that can be exposed without risk of intra- or postoperative complications. Finally, the plate, can be fixed in its proper position by microscrews. The small reinforcement plate is not only used for increasing strength and rigidity of the fixation system but also determines the position of the drilling holes and proper angulation of the long positional screws.

To have the necessary mechanical properties, such a thin construction should be made only of metal (titanium is preferable), which can be manufactured through direct laser metal sintering (DMLS) with a precision of 0.8 mm. Before beginning research on clinical efficacy and potential limitations for such an approach, we simulated the plate’s biomechanical behavior through finite element analysis (FEA) as presented in the article.

FEA is a computational technique used to model the mechanical behaviour of structures, including biological tissues, which can measure parameters that cannot be directly assessed in vivo (such as the internal stress and strain of bones and fixation hardware). FEA can be considered a powerful instrument for preclinical estimation as well as comparison of various fixation techniques and optimization of surgical approaches in facial fractures; it has been successfully used in numerous studies to investigate distribution stresses and strains in screw and plate ostheosynthesis [37]. Recent FEA simulations demonstrated that conventional two-screw osteosynthesis of the condylar head had significant biomechanical advantages over the other fixation techniques [9, 23]. However, these studies analysed only typical B-type fractures (i.e. corresponding to the type p fractures of the present study) rather than addressing clinically frequent multifragmented fractures or biomechanically unfavourable fractures, such as those with a compromised cortex of the lateral condylar stump [25]. We also found no experimental reports regarding the possibilities for reinforcement of the two-screw fixation system with patient-specific implants or conventional mini- or microplates.

Previous studies have demonstrated that FEA model accuracy is increased as model complexity and the number of finite elements increases [9, 21, 23, 32]. The number of nodes and elements in our models was sufficient for proper representation of the complex geometry of the mandibular bone and its inner structure. In previously reported models of the human mandible, the number of elements ranged from 22,986^10^ to 1.5 million [32]. In this study, special attention was given to the accurate simulation of muscular forces in various loading conditions. In previous studies regarding the finite element simulation of mandible loading, authors assigned anywhere from two to nine mandibular muscles with simulations of different occlusal contactsor forces [9, 32, 36]. In our study, the application of constraints and muscular loads was performed according to the concepts described by Korioth and Koolstra, as is common in more recent FEA studies [28–30].

With these parameters, the model provided a close approximation to real clinical conditions and results, comparable with previously published data.

The material properties used in the models were taken from the literature and based on the direct measurements of the intact bones. For such a simulation one should be aware of the fact than the main mechanical constants of the cortical and spongious bone may vary significantly in different individuals and depend on the method used for their estimation in experimental studies. The reported values of the Young’s modulus for cortical bone used for FE modeling is from 4 to 20–22 GPa, and for spongious bone from 0,05 to 1,5 GPa. The trauma itself and the posttraumatic bone remodeling also influence the mechanical properties of the condylar head, but there is lack of information concerning this issue due to significant limitations for the direct measurements in the human beings.

At the same time the models, created in our study, give and adequate representation of the general patterns for stress and strain distribution. They can be also used for comparison of the different fixation techniques and prediction of changes/errors associated with altered mechanical properties if lineally elastic behavior of the system is assumed.

The generated data on stress and strain distribution inside the bone and screws, as well as the total deformation values, corresponded to the previous research on this topic. The post-processing of the FEA models demonstrated that stress was transmitted through the screws to the medial fragment and that adequate rigidity was achieved at normal chewing, with no signs of stress concentration higher than the ultimate level. However, if maximal force of the muscular contracture was applied, the model revealed areas of excessive stress concentration appearing near the screw holes, predominantly at the cortical layer of the lateral stump. Von Mises stress concentration is an important indicator in the assessment of bone remodeling; Sugiura et al. indicated that stress exceeding 50 MPa in bone can lead to bone resorption [38]. In our simulations, the maximum von Mises stress exceeded 50 MPa in conventional two-screw fixation at maximal loading, thus demonstrating the possible risks of this method in unfavourable biomechanical conditions. Excessive strains may lead to failure of fixation due to bone resorption, cracks, fragmentations and loosening of the screws.

In the models with patient-specific reinforcement, plate stress and strain distribution patterns were the same – however, their values were 2–10 times lower than in the conventional. Stress values with patient-specific reinforcement were less than 50 MPa even in cases with maximal muscular forces. The rigidity of fixation also increased and was much closer to normal jaw deformability.

Although application of the reinforcement plate had little, influence on the maximal stress values in the titanium hardware itself (especially inside the screws), these values were less than ultimate stress for the titanium even in maximal loading conditions, the clinical data. Excessive stress developed only at the thread edges, which can undergo plastic deformation without fixation failure.

As was reported in recent studies, trauma resulting from extensive exposure of the condylar head fracture and application of micro- or miniplates often causes scar-induced limitation of postoperative translation in the joint [12, 13, 39] and even development of postoperative fibrous ankylosis in at long-term follow-up [3]. In the current study, the patient-specific plate has a special design that minimizes the need to detach the capsule from the lateral pole area. This aspect, along with the low bone strain and minimum condylar head displacement demonstrated in the current FEA models, suggests that screw fixation with patient-specific reinforcement plate (PSRP) will perform best in fixation of condylar head fractures in certain biomechanically unfavourable fractures.

This study has some limitations. As with any simulation approach, simplifications and assumptions were made in the models: the bone was considered to be homogenic and isotropic within one material type; only the static loading conditions of anterior biting were reproduced; a linear elastic solution was applied, and others. In reality, the biomechanical properties and behaviour of the bone is more complicated and may significantly change in the injured areas. However, authors in many studies use such simplifications and obtain results which correlate with clinical data and experiments in cadaveric bone. In post-processing, we also ignored the regions of artificially high strain that some authors consider to be errors of numerical simulations. Additionally, it is impossible to predict the reaction of soft tissue to installation of the construction as well as clinical peculiarities and limitations for application of the proposed method.

Thus the results of a simulation should be critically judged any clinical recommendations should be made with proper caution. The further work is also needed to validate the results in both experimental and clinical studies.

## Conclusion

The findings of the current study suggest that reinforcement of conventional two-screw fixation of condylar head fractures with a small patient-specific plate acting as a washer may have significant benefits in unfavourable biomechanical conditions. PSRP provides a decrease in equivalent stresses in the bone up to 2–10 times and an increase in fixation rigidity of up to 1.25–3 times compared to the conventional technique. Further comparative mechanical and clinical studies are needed to confirm this observation.

## Data Availability

The datasets used and/or analysed during the current study are available from the corresponding author on reasonable request.

## Abbreviations

FEA: Finite element analysis
ORIF: Open reduction and internal fixation
TMJ: Temporomandibular joint
CAD: Computer-aided design
CAM: Computer-aided manufacturing
PSRP: Patient-specific reinforcement plate
CT: Computed tomography
